# Enhanced remediation of contaminated aquifers by circulating flow field: Laboratory sandbox with quantitative analysis

**DOI:** 10.1016/j.isci.2026.116237

**Published:** 2026-07-09

**Authors:** Huiyang Qiu, Ziwen Zhou, Lei Wang, Jing Hua, Chicgoua Noubactep, Yang Song, Yizhi Yuan, Rui Hu

**Affiliations:** 1 School of Earth Sciences and Engineering, Hohai University, Nanjing 211100, China; 2 Nanjing Institute of Environmental Sciences, Ministry of Ecology and Environment of the People’s Republic of China, Nanjing 210042, China; 3 Department of Earth and Environmental Sciences, University of Waterloo, 200 University Ave. W., Waterloo, ON N2L3G1, Canada

**Keywords:** Chemistry, Engineering, Environmental science

## Abstract

Soil and groundwater contamination are escalating due to anthropogenic activities, necessitating cost-effective remediation technologies. This study integrates laboratory experiments and numerical simulations to investigate extraction-injection circulation enhancing remediation agent transport in heterogeneous aquifers. A 2D irregular heterogeneous sandbox model was developed, with high-precision flow field inversion via hydraulic tomography using 20 tests (*R*^2^ > 0.89). Extraction-injection experiments varied well positions, injection modes, and agent concentrations. Remediation agent migration and distribution under flow were monitored through 36 sampling ports. Based on the advection-dispersion equation, the spatiotemporal agent distribution agreed with measurements (*R*^2^ > 0.71), validating reliability. Numerical modeling revealed that extraction-injection well elevation differences and pumping rates influence migration efficiency. Empirical equations quantified relationships between these factors and temporal/spatial agent distribution at extraction wells. This work elucidates recirculation-enhanced transport mechanisms, bridging high-precision groundwater modeling with remediation optimization. The findings provide a theoretical framework for designing efficient remediation systems at contaminated sites.

## Highlights

• Hydraulic tomography characterizes heterogeneity to guide remediation’s efforts
• Distribution of agents due to extraction-injection circulation is better quantified
• Remediation processes by injected agents in heterogeneous aquifers are clarified
• Influencing factors and transport dynamics of injected agents are correlated

## Graphical abstract

**Figure:**
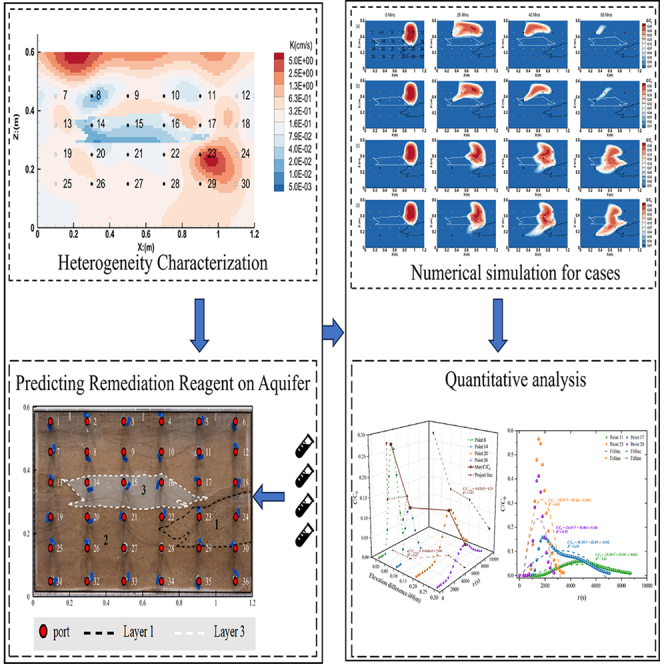

## Introduction

Groundwater, the most important “invisible” freshwater resources on Earth, is currently experiencing both qualitative and quantitative degradation due to overexploitation and pollution from agricultural, communal, domestic, and industrial human activities.^1^^,^^2^^,^^3^ This sad situation has prompted scientists to seek affordable, applicable, duplicable, and efficient solutions to treat polluted groundwater. Relevant applicable technologies include: (1) bioremediation, (2) *in situ* air sparging (IAS), (3) *in situ* chemical oxidation (ISCO), (4) pump and treat (P&T), and (5) permeable reactive barriers (PRBs). The ideal technology should be cost-effective, environment friendly, efficient, and be able to remove all types of contaminants and contaminant groups.^4^

P&T has been the most common groundwater remediation method for many decades.^5^^,^^6^^,^^7^ It has played a critical role in controlling contaminants and water management.^8^ However, in most cases it cannot reduce the contaminant level below the regulatory standards because of the difficulty on reagent spreading, while having high pumping costs for long periods.^9^ On the other hand, bioremediation and ISCO are mostly used to treat organic pollutants and cannot remove heavy metals.^10^ For all these reasons, PRBs and *in situ* treatment zones are the most suitable groundwater remediation methods.^11^ However, the invisible underground is naturally heterogeneous. And the operating mode of many PRBs implies that the heterogeneity heightens with time.^7^ This makes hydraulic tomography (HT) an important tool to assist both characterization and remediation actions.^12^

Transforming pollutants (e.g., inorganic and organic compounds and heavy metals) to less toxic species by chemical reagents is a reasonable remediation method. Variables impacting the remediation efficiency (reaction kinetics and extent of remediation) include: (1) activator used,^13^^,^^14^ (2) contact time,^15^ (3) oxygen content,^16^ (4) pH value,^17^^,^^18^^,^^19^ and (5) temperature.^20^^,^^21^ The chemical reaction is critical to remediation, while delivery of reagent has a key role equally. The objective of reagent delivery is to provide sufficient reagent (concentration and volume) and having adequate residence time to meeting the requirements of the chemical reactions.^22^ With the effect from heterogeneous aquifer, the injection of remediation reagent would lead to the uneven distribution of reagent.^23^

A classic method is that stimulating hydraulic behaviors for enhancing reagents delivery after injecting reagents. For instance, Pac et al. presented a constant head injection for enhanced reagent delivery.^24^ Wang et al. enhanced delivery of remedial reagents in low-permeability aquifer by groundwater circulation well (GCW).^25^ Lubrecht used horizontal directional drilling as a new technique for transporting remediation reagents.^26^ Zhang et al. enhanced groundwater remediation efficiency through integrating P&T system and GCW.^27^ With the enhancing effect from hydraulic behaviors, it is important for having the most accurate prediction of reagents transport on groundwater flow field. Groundwater modeling provides a fundamental solution for solving this issue, which is often based on numerical simulation of classical advection-dispersion model.^28^^,^^29^^,^^30^ However, it is still a challenge for predicting complex solute transport on groundwater with accurate precision. Many studies found that the prediction of solute transport has obvious space with observation because of the complex geological structure,^31^ density of reagent,^32^ temperature,^33^ and so on.

Parameter estimation of medium is one of keys for predicting solute transport on heterogeneous aquifer.^34^ After 25 years field research on Columbus Air Force Base in Mississippi, Zheng et al. found that the advection-dispersion model is inadequate for predicting solute transport on a heterogeneous field site.^35^ The advection-dispersion model used kriging for heterogeneity characterization maybe one of reasons, which has been found to have negative effects on predicting than using highly parameterized model. HT is a method for characterizing aquifer heterogeneity. Based on better abilities on calibrating head data and predicting hydraulic behaviors, it has better performance than kriging or other traditional methods proved by many studies.^36^^,^^37^^,^^38^^,^^39^ However, there are little studies based on attempted HT in the predicting of contaminant remediation.

Based on the characterization of HT, prediction of groundwater modeling has found obvious improvements. For instance, Jiménez et al. coupled HT with advection-dispersion model for predicting solute transport in a heterogeneous aquifer with better matching of observed data.^40^ Zhao et al. found that HT results predicted both migrating plumes and breakthrough curves with good performance.^41^ However, these studies focus on the performance of model validation, while they neglect the further study on impacting factors of enhancing tracer spatial distribution. Zhang et al. utilized high-resolution aquifer characterization for discussing influences and performance of parameters on flow field.^42^ Yet, it only involves synthetic aquifer, which lacks the data from real world. To our best knowledge, there is no study that involves heterogeneity characterization with high-resolution for identifying the influence factor of the reagent spatial distribution with calibration of laboratory data.

In this study, we apply the high-resolution *K* fields obtained by HT in prediction of the spatial distribution for several tracer injections. Objectives of this study include: (1) investigating the usefulness of heterogeneity characterization from HT based on data from laboratory experiments with a layered-structure aquifer; (2) identifying the enhancement effect from hydraulic stimulation for reagent delivery; (3) assessing influence factors of reagent transport on heterogeneous aquifer based on groundwater modeling considered high resolution characterization from HT. We first obtain the heterogeneity characterization based on HT and geological zonation model in a multi-layered laboratory sandbox with known stratigraphy. Then, based on the reconstructed *K* fields, groundwater modeling with classical advection-dispersion equations (ADE) was used to simulate the process of tracer injection and transport under conditions the same with laboratory experiments. Simulated drawdown data and concentration data were compared with observed data for evaluating model accuracy. At the end, this study discusses influencing of wells location and pumping rate based on a well calibrated simulation model predicting results. This study provides further insight into coupling groundwater modeling with high-resolution parameters, which is important for geo-scientists to have an understanding for predicting remediation processes with groundwater modeling.

## Result

### Permeability distribution and flow field dynamics from laboratory experiments

**Figure 1:**
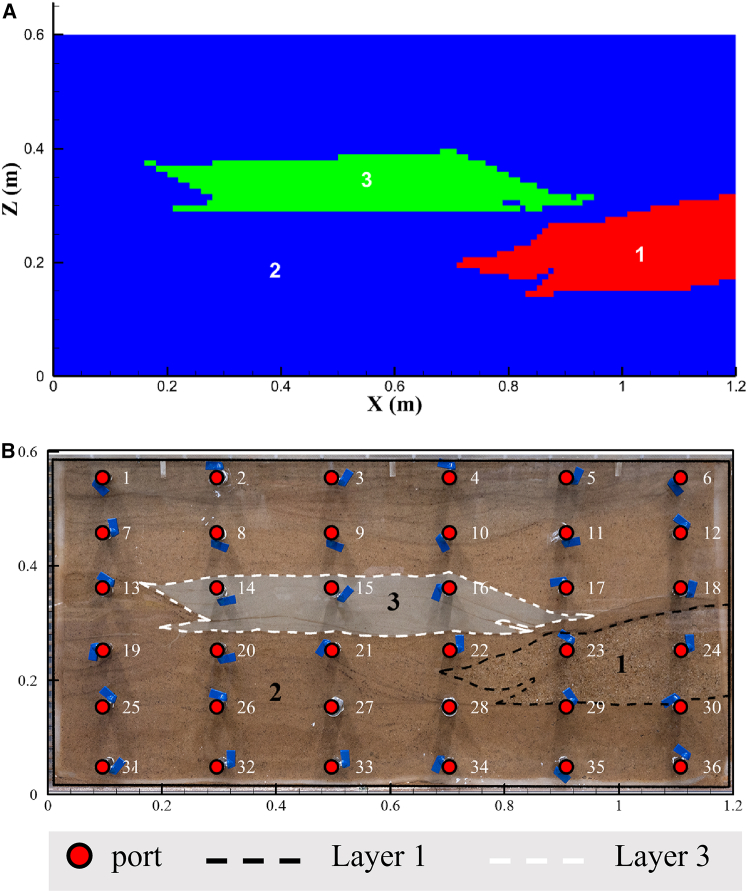
Schematic representation of the heterogeneous aquifer sand tank assembly, with 3 distinct aquifer and 36 instrumented access ports (A) Geological structure map; (B) photograph on the laboratory (Qiu et al., 2025)

**Figure 2:**
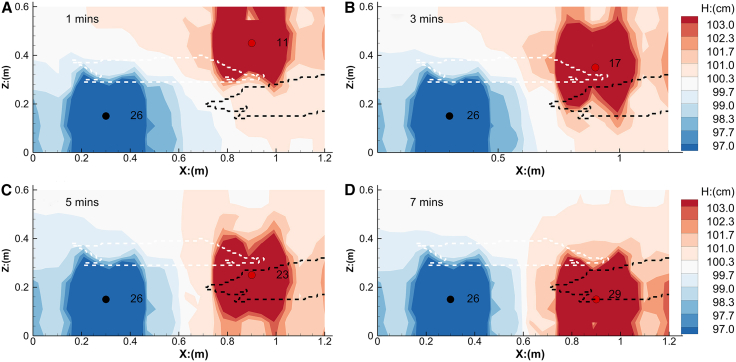
The water-head distribution diagram with time of laboratory excavation-injection experiment (test 4)

Interpolation of pressure measurements at different ports in the sandbox experiment yielded the hydraulic head distribution (Figure 1), as illustrated by test 4, which consisted of 4 injection tests. Equal remediation agents’ volumes were injected at a constant rate through four vertically arranged ports (11, 17, 23, and 29), and the head distribution at the end of each test is shown in Figure 2. The results demonstrate highly similar spatial head distribution patterns across all 4 tests: the head values progressively decrease from the highest (110 cm) near the injection ports on the right side to the lowest (90 cm) near the extraction port at the lower left corner, consistent with a source (injection)-sink (extraction)-driven flow mechanism. The contour of water table exhibits a quasi-stratified distribution with relatively uniform head gradients (indicated by isopleth density) and no significant localized distortions or concentrated flow paths, suggesting the absence of pronounced preferential flow within the medium. The negligible influence of vertical injection port positions on the overall flow pattern indicates their weak impact on the regional seepage field, while simultaneously highlighting the dominant role of the extraction port, whose pumping-induced cone of depression extends over the lower right section of the sandbox, establishing a relatively stable flow field and contributing to pattern convergence. Additionally, the scale effect of the sandbox likely restricted vertical flow differentiation, further homogenizing the effects of vertical position variations.

### Spatial and temporal distribution of tracer concentrations from laboratory experiments

**Figure 3:**
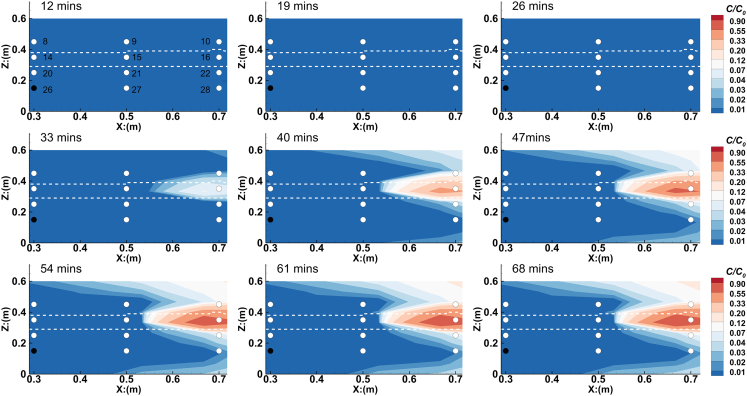
The concentration distribution diagram with time of laboratory excavation-injection experiment (test 2)

The concentration distribution map was obtained by interpolating concentration measurements from different ports in the sandbox experiment (Figure 3). In test 2, at nine different ports (10–68 min), the tracer consistently migrated directionally from the injection port toward the extraction port (black), forming a distinct “tongue-shaped” advancing front. In the early stage, the tracer slowly migrated leftward from the injection port but had not yet reached the farthest right column of observation ports, thus going undetected. From the middle stage, the tracer front reached the farthest right column, elongating the concentration front into a “narrow tongue” porting toward the extraction port, with an increasing concentration gradient in that direction. Over time, the tracer concentration in this column gradually rose (from 0.04 to 0.07 to 0.55–0.90), indicating the movement of high-concentration regions into this area. In the late stage, the position and magnitude of the concentration front remained largely unchanged indicated tracer transport stabilized, with only a slight expansion of the high-concentration zone. This behavior aligns with both the properties of the low-permeability layer (layer 3) and the re-equilibration of the seepage field in the sandbox after tracer injection. In the initial phase of tracer migration, the predominant direction of movement was from the injection port toward the extraction port (lower left), confirming the strong control of the pumping port on solute transport pathways in homogeneous porous media, where the sink effect induced by pumping dictated solute movement. Once the tracer migrated into layer 3, it exhibited primarily advective transport, reflecting the diminished influence of pumping in low-permeability zones. Simultaneously, the tracer front maintained a port-defined “tongue-shaped” morphology, elongating along the pumping direction, indicating advection as the dominant mechanism of solute transport, with minimal dispersion effects.

In contrast, the concentration field in test 3 exhibited more complex evolution, generally progressing through three distinct phases. During the early stage, the tracer injected at the injection port migrated relatively swiftly toward the lower-left direction under pumping influence, with the first detection already reaching the farthest right column of observation ports. In the intermediate phase, under the combined effects of the extraction port, injection port, and the low-permeability layer (layer 3), the tracer initially underwent advective transport via preferential flow paths above the low-permeability zone before being drawn downward toward the extraction port by the dominant sink effect. Concurrently, the enhanced hydraulic gradient between injection and extraction ports, resulting from continuous injection, facilitated gradual tracer penetration through the low-permeability layer toward the extraction port. The late stage saw continuous freshwater injection simultaneously intensifying the hydrodynamic field and completely flushing out residual tracer at the injection port, effectively creating a “zero-concentration source” at the injection port. While the extraction port exhibited progressively rising tracer concentrations (from <0.01 to 0.04–0.07) due to cumulative solute migration before ultimately returning to initial conditions under sustained pumping. Overall, test 3 developed an elongated, sinuous contaminant plume extending from injection to extraction ports with extensive coverage, displaying irregular diffusion patterns with serrated edges that demonstrated enhanced mechanical dispersion from continuous injection. The distinctly curved plume boundaries observed during early- and mid-stages indicated strong dispersive effects, whereas the later-stages directional extension revealed advection-dominated transport mechanisms, their interplay resulted in non-monotonic concentration attenuation along the migration path (mid-section > end port).

In test 1, since the tracer was injected at port 29, its migration was less affected by the low-permeability layer, exhibiting predominantly advection-driven transport controlled by pumping effects with significantly higher velocity than test 2 (Figure S1). The extraction port concentration underwent a similar gradual increase followed by decrease as observed in test 3. In test 4, tracer injected at ports 11 and 17 underwent advective transport similar to test 2, slowly migrating through the low-permeability layer, while tracer injected at ports 23 and 29 was dominantly influenced by pumping effects, moving relatively faster through the high- and medium-permeability zones beneath the low-permeability layer toward the extraction port (Figure S2). These latter two injection ports functioned similarly to the continuous injection using water in test 3, accelerating tracer movement within the low-permeability layer to some extent. Upon cessation of tracer injection from all four ports, hydrodynamic driving forces weakened and tracer migration within the low-permeability layer returned to slow conditions, ultimately demonstrating upward movement of high-concentration zones.

Comparative analysis of these four experimental results clearly demonstrates that extraction ports serve as the core engineering measure for controlling contaminant (remediation agents) transport in aquifers, while continuous water injection substantially alters contamination distribution patterns through dual mechanisms of “source cleansing and solute driving.” These phenomena provide crucial reference values for contamination remediation strategy design.

### Permeability distribution and flow field dynamics from simulation under recirculating flow

**Figure 4:**
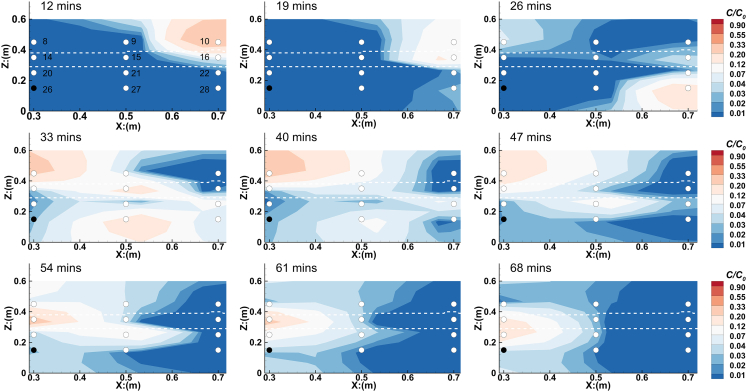
The concentration distribution diagram with time of laboratory excavation-injection experiment (test 3)

**Figure 5:**
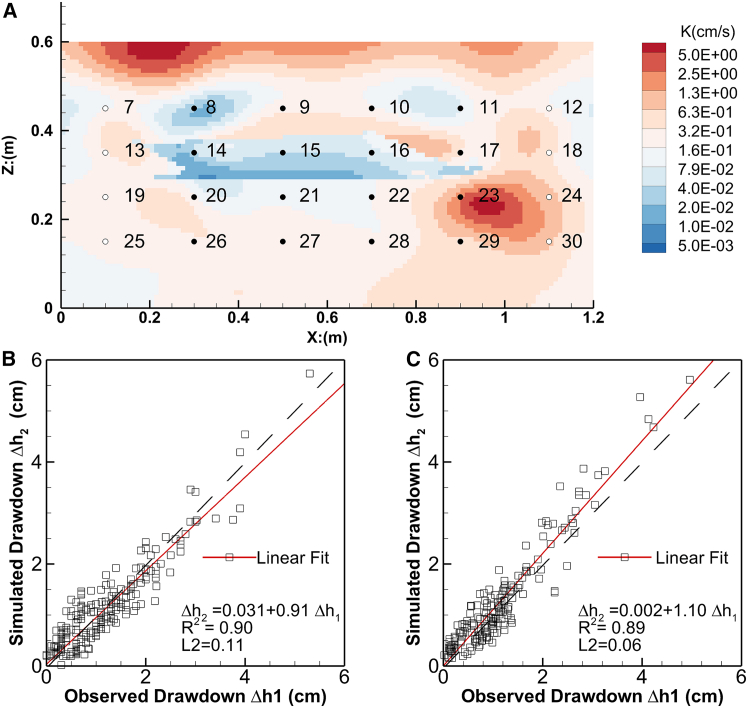
Results of hydraulic chromatography (A) Inversion results of aquifer permeability coefficient, (B) calibration, and (C) validation. Squares represent data points, black dashed line represent the 45° line between sim. data vs. ob. data.

Following the methodology described in this section, the HT approach was employed to invert model parameters using data from 24 effective sensors across different experimental groups. The model calibration dataset incorporated 8 pumping tests and 4 extraction-injection tests, while validation data primarily came from an additional 8 pumping tests. The HT inversion results distinctly revealed the macroscopic spatial zonation characteristics of permeability coefficients in the sandbox, successfully identifying three principal zones corresponding to the actual physical model (Figures 1 and 4): the high-permeability zone (*k* ≈ 0.3–0.4 cm/s) shown as a red area in the lower right corner, the low-permeability zone (*k* ≈ 0.003 cm/s) depicted as a dark blue region in the upper left corner with boundary contours highly consistent with the actual sandbox structure, and the continuous yellow-green intermediate-permeability zone (*k* ≈ 0.05–0.1 cm/s) in the central section (Figure 5). Numerically, the inverted value for the low-permeability zone (0.003 cm/s) closely matched the true value (0.00351 cm/s). Actual sharp interfaces (e.g., between layer 1 and 2) appeared as smoothed transitional zones (yellow to blue gradation) in the inversion, exposing resolution limitations, with an erroneously identified high-permeability patch (*k* ≈ 0.2 cm/s) in the upper right corner possibly due to boundary flow interference.

Overall, the HT inversion achieved accurate identification of macroscopic zoning and critical barriers while effectively characterizing the aquifer framework structure, consistent with the high correlation (*R*^2^ = 0.9) between predicted and observed head changes at various ports in Figure 5B. The similarly strong correlation (*R*^2^ = 0.89) in the validation set confirmed the absence of overfitting in this inversion model (Figure 5C).

### Simulated migration and spatial-temporal distribution of tracer under recirculating flow

**Figure 6:**
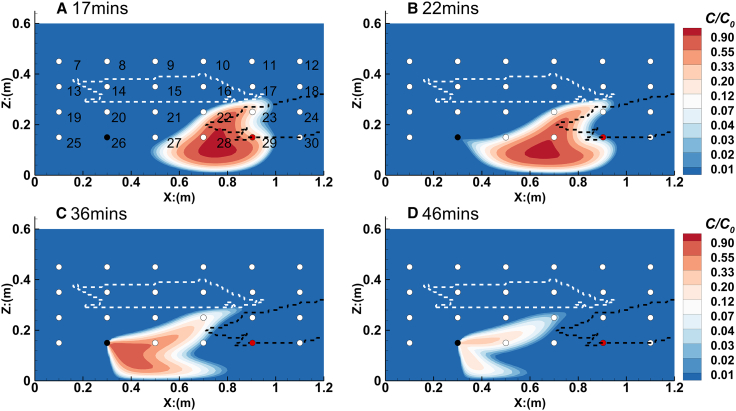
The concentration distribution diagram with time of modeled extraction-injection experiment (test 1)

**Figure 7:**
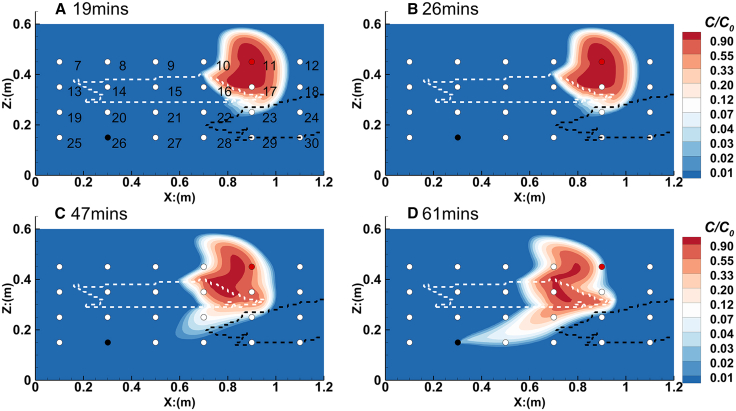
The concentration distribution diagram with time of modeled extraction-injection experiment (test 2)

Using the sandbox parameters derived from the previous inversion, simulations of concentration fields were conducted for different experimental groups. The inversion results for test 1 successfully reproduced the core transport patterns of the tracer within the sandbox: the tracer primarily migrated along the path from the injection port to the extraction port (Figures 6 and S1), with the overall curved morphology largely consistent with physical experimental observations. The positions of high-concentration zones (*C*/*C*_0_ ≈ 0.6) in the central section also showed good agreement, confirming the model’s reliability in capturing major solute transport pathways. Similarly, the inverted results for test 2 matched the physical experiments in terms of both the bending direction of the tracer plume’s main axis and the locations of high-concentration zones (Figures 3 and 7). Results from both tests clearly demonstrate the significant influence of aquifer heterogeneity on tracer transport, revealing striking contrasts between rapid migration through medium-high permeability zones and delayed movement in low-permeability layers.

**Figure 8:**
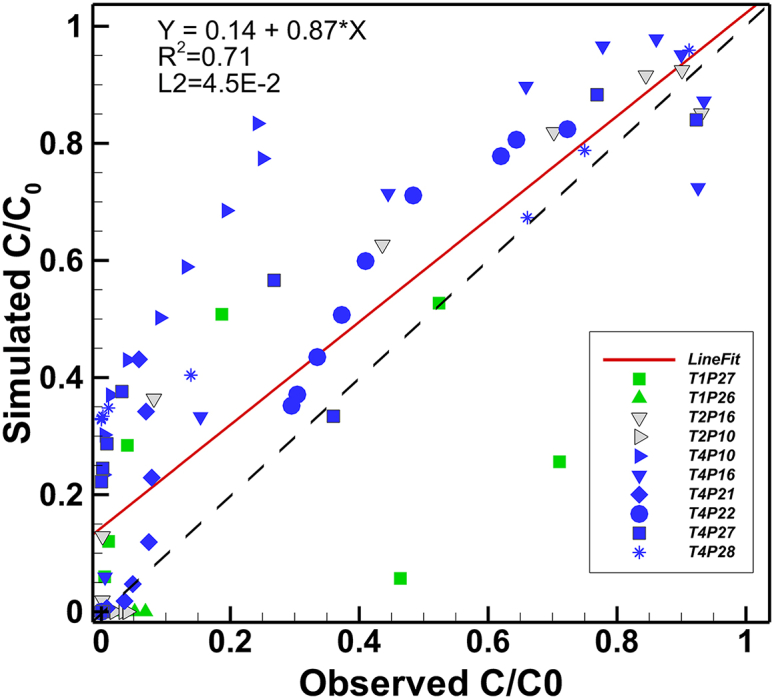
Comparative scatterplot of predicted and observed solute concentrations

Limitations do exist in the inversion model: inherent smoothing effects lead to excessively smoothed plume edges, some deviations appear near source/sink ports, and the model performs relatively poorly in capturing rapid tracer transport, particularly during water injection (Figure S3). Nevertheless, this concentration-field inversion technique demonstrates robust performance in reproducing macroscopic transport characteristics (including plume axis orientation and high-concentration zone locations), sufficiently meeting preliminary pollution risk assessment requirements. Regarding simulation accuracy, the fitting between calculated and measured concentrations at monitoring ports across different test groups (Figure 8) yields *R*^2^ values of 0.71 for tests 1, 2, and 4, indicating excellent agreement and confirming the model’s capability for precise simulation of tracer transport pathways and concentration field distributions under various experimental conditions.

## Discussion

### Enhanced migration effects in extraction-injection circulating flow field

Analysis of tracer spatial distribution patterns from both laboratory experiments (Figure 3) and numerical models (Figures 6 and 7) across different experimental groups revealed that test 1 exhibited the poorest tracer transport performance, whereas test 3 significantly improved tracer distribution and extended its coverage through continuous water injection. Test 4’s multi-layer injection approach notably increased tracer concentration near the injection ports, while monitoring locations at greater distances consistently showed lower concentrations.

These observations demonstrate that hydraulic-driving forces play a crucial amplification role in tracer transport. Under enhanced hydraulic gradients, tracers with identical initial concentrations can migrate substantially farther. This phenomenon is similar with a previous study,^43^ which found influence radius of wells get larger with the hydraulic gradients.

Previous study of Qiu et al. found that the distribution of residual contaminants shows the trend of uniform distribution after remediation by coupling GCW and ISCO. Positive effect of using GCW can be better explained by this study. At distant monitoring ports, observed concentrations under hydraulic enhancement may even exceed those achieved through higher dose stratified injections. The phenomenon is supported by a pervious study which found the flow field enhances the delivery of reagent.^25^ This highlights the vital importance of circulation flow fields for pollution remediation. By controlling hydraulic conditions to simultaneously meet both temporal and concentration requirements for reagent delivery to target zones, remediation efficiency can be substantially improved.

### The main influencing factors of enhanced migration in extraction-injection circulating flow field

#### Depth of the extraction port

Existing studies have investigated the influence mechanisms of remedial reagent concentration and circulation operation mode on enhanced reagent transport,^25^ yet the selection and impact of well spatial positioning have rarely been discussed. To investigate the influence of different extraction port depths at the same location on enhanced migration effects, a series of tracer transport simulations (case 1) were conducted based on the established numerical model with proven effectiveness (details of parameters setup and phenomenon analysis can be seen in supplemental information).

**Figure 9:**
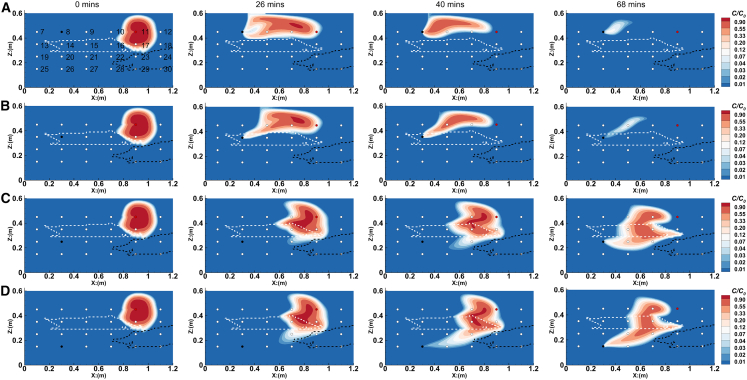
Simulation result for case 1 using various extraction ports (A) Port 8; (B) port 14;(C) port 20; and (D) port 26. Red dots represent injection ports, and black dots represent extraction port.

The simulation results clearly demonstrate that different excavation port depths at the same location significantly affect tracer diffusion and enhanced migration effects (Figure 9). The excavation port depth not only affects diffusion speed and range but also the morphology of tracer spread. Beyond being controlled by excavation port depth, these simulation results were also influenced, albeit not dominantly, by aquifer heterogeneity, as evident from comparisons between port 8, 14, and 20 (Figures 9A–9C). In conclusion, at the same port location, the smaller depth difference between the excavation and injection port, the faster the tracer diffuses, the larger the spread, and the more uniform the distribution shape. With a previous study supported, it is meaningful for considering the enhancing migration of reagents.^44^ The enhancement of reagent can have a positive effect for improving *in situ* remediation efficiency.

Additionally, heterogeneous aquifer properties exert some influence on tracer migration rates. These findings hold substantial significance for optimizing tracer transport behavior and improving detection efficiency. By rationally selecting excavation port depths, migration pathways can be better controlled, ensuring both remediation efficiency and effectiveness at the site.

#### Depth of the injection port

**Figure 10:**
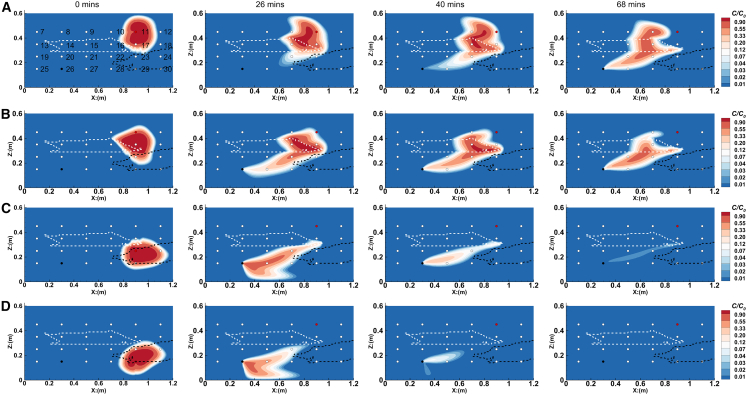
Simulation result for case 2 using various injection ports (A) Port 11; (B) port 17; (C) port 23; and (D) port 29. Red dots represent injection ports, and black dots represent extraction port.

Similarly, a series of tracer transport simulations were conducted using the port-established and highly effective numerical model to investigate the influence of different injection port depths at the same location on enhanced migration (case 2) (the details are in supplemental information). The simulation results distinctly reveal that different injection port depths at the same location significantly influence tracer diffusion and enhanced migration. The injection port position governs not only diffusion speed and range but also the shape of tracer spread. Analogous to the variable-pumping-port tests, the variable injection port simulations were also moderately influenced by aquifer heterogeneity, as evidenced by comparisons among ports 17, 23, and 29 (Figures 10B–10D). In summary, tracer diffusion accelerates and broadens with more uniform morphology as the injection port depth approaches the pumping port depth.

Notably, the initial concentration distributions differed markedly between variable-pumping-port (case 1) and variable-injection-port (case 2) tests. In case 1, initial distributions were consistent across all pumping ports, with tracers diffusing radially from the injection port (the high-concentration zone) and exhibiting leftward migration tendencies. In contrast, case 2 displayed pronounced initial disparities among injection ports, directly linked to formation heterogeneity. Crucially, combining results from both test types clarifies that tracer migration rates depend not on the absolute depths of pumping or injection ports but on their relative depths. Specifically, smaller relative depths (i.e., closer vertical separation) between pumping and injection ports correlate with faster diffusion, larger spread, and more uniform morphology. This phenomenon is consistent with experience on field study.^45^ Hence, strategic selection of injection and pumping port positions thus enables precise control over tracer migration pathways, enhancing both the accuracy and efficiency of soil and groundwater remediation efforts.

#### Pumping rate

Existing field-scale studies have demonstrated enhanced contaminant removal from groundwater through recirculation flushing,^45^ yet the absence of quantified relationships between pumping/injection rates and remediation efficacy has constrained the implementation of precision remediation at contaminated sites. A series of tracer transport simulations were conducted using the port-established and high-performance numerical model to investigate the influence of different pumping rates on enhanced migration effects (case 3) (the details are in supplemental information).

As the pumping rate increased from 8 to 32 mL/s, the tracer’s diffusion range expanded overall but the expansion exhibited nonlinear behavior (Figure S5). Higher pumping rates increase hydraulic gradients, intensifying advective transport and enabling the tracer to overcome permeability barriers. In contrast, permeability variations dictate local flow velocities and preferential migration paths by altering effective porosity and hydraulic conductivity. The simulation result is consistent with a previous study which proves the enhancement of hydraulic effect.^25^

Low-permeability zones, due to high capillary resistance and limited effective flow paths, significantly slow tracer front propagation, resulting in lower accumulated concentrations. Intermediate-permeability zones form transitional diffusion zones under elevated hydraulic gradients, while high-permeability zones consistently dominate the primary migration pathways. It is noteworthy that as pumping rates increase, the capture efficiency of high-permeability zones diminishes (plume widening reduces core concentrations), while contributions from intermediate- and low-permeability zones gradually rise. It means that the strong heterogeneous aquifer could be a reason for back diffusion phenomenon on the remediation scenario.^46^^,^^47^^,^^48^ Illman et al. found tracer migration in heterogeneous aquifers affected by permeability heterogeneity.^49^ This study indicates that tracer migration shaped simultaneously by pumping intensity and permeability heterogeneity.

#### Quantitative analysis

The previous analyses demonstrate that the regional tracer concentration field undergoes dynamic adjustments in response to variations in parameters. However, the temporal evolution of concentrations at key monitoring points, particularly extraction wells, holds greater practical significance for remediation applications. Therefore, systematic analyses were conducted to characterize the evolution of relative tracer concentrations (*C*/*C*_0_) at pumping wells in heterogeneous aquifers with respect to migration time (*t*), elevation difference (*δ*H) between pumping and injection wells, and pumping rate (*v*) (Figure S6).

Figure S6A reveals distinct concentration response patterns across monitoring points for case 1, which primarily stem from the coupled effects of *δ*H and spatial positioning. Notably, all breakthrough curves followed a consistent pattern: rapid ascent to peak concentration followed by slow decay (Figure S6B), though decay rates varied spatially due to permeability-dependent tracer retention. Multi-linear regression yielded empirical time-concentration expressions with satisfactory goodness of fit (*R*^2^ > 0.6, mean >0.7), enabling predictive applications; the outlier poor fit for port 20 likely relates to stratum 3’s low permeability. It reveals an inverse correlation between *δ*H and both concentration magnitude and time to peak (solid line, Figure S6A). With strong linear trends emerging between *δ*H versus time to peak (*R*^2^ = 0.87) and *δ*H versus peak concentration (*R*^2^ = 0.67). These high-fidelity empirical relationships, considering dominant transport mechanisms and permeability heterogeneity, provide robust tools for spatiotemporal concentration prediction.

(Equation 1) CC0max=−0.65δH+0.24.

(Equation 2) t(CC0max)=11400δH+2100.

Similarly, distinct curve morphologies emerge: lower injection wells yield steep ascents with sharp maxima, whereas higher wells produce broad plateaus with blunt peaks for case 2 (Figure S6D). Fitted concentration-time empirical expressions demonstrate reasonable predictive capability (*R*^2^ > 0.55, mean >0.65) for pumping well concentrations. It confirms linear relationships between *δ*H and both time to peak (positive correlation) and peak concentration magnitude (negative correlation). These trends reflect physical retardation and hydrodynamic dispersion effects along extended migration pathways. High-fidelity empirical relationships (0.71 and 0.68) quantitatively capture these spatiotemporal distribution patterns. Proximal injection wells coupled with high-permeability pathways enable rapid tracer advection (short transit times, limited dilution), whereas distal wells force circuitous routing through low-permeability zones, extending migration paths while enhancing diffusion-driven attenuation (50% *C*/*C*_0_ reduction). These mechanisms provide critical insights for predicting contaminant trajectories and remediation timelines in heterogeneous aquifers.

(Equation 3) CC0max=−1.46δH+0.52.

(Equation 4) t(CC0max)=10350δH+885.

Parallel analysis of case 3 demonstrates curves from different rate groups exhibit significant spatial differentiation: High-rate clusters (*v* ≥ 24 mL/s) form steep cones (*C*/*C*_0_ > 0.75) at short timescales (*t* < 3,000 s), while low-rate groups (*v* ≤ 12 mL/s) spread as subdued platforms (*C*/*C*_0_ = 0.3–0.5) beyond 6,000 s (Figure S12E). Robust concentration-time models (*R*^2^ > 0.75, mean >0.8) support operational predictions.

(Equation 5) CC0max=−0.0007v+0.064.

(Equation 6) t(CC0max)=−294.18v+10603.

XY-plane projections (*R*^2^ = 0.84) confirm that increased *v* reduces time to peak by enhancing hydraulic gradients and preferential advection. Conversely, XZ-plane trends (*R*^2^ = 0.94) reveal paradoxical peak suppression at higher rates, accelerated transport causes premature concentration decline, potentially compromising remediation efficacy. These quantifiable rate-dependent spatiotemporal patterns establish a verifiable framework for optimizing extraction-injection parameters in heterogeneous groundwater systems.

Against the backdrop of growing demands for more effective, economical, applicable, and safer remediation technologies in contaminated site restoration, this study systematically evaluates the transport mechanisms of remediation agents in heterogeneous aquifers through integrated laboratory experiments and numerical simulations. HT was employed to characterize aquifer heterogeneity, demonstrating its practical utility in predicting contaminant migration. By investigating the enhanced transport of remediation agents under extraction-injection circulation flow conditions in heterogeneous aquifers, this research provides theoretical foundations and practical guidelines for optimizing remediation strategies in soil and groundwater pollution control. The key findings are as follows.

(1) A two-dimensional irregular heterogeneous sandbox model (*k* = 0.003–0.4 cm/s) was developed, enabling high-precision inversion of heterogeneous media via HT method (validation *R*^2^ > 0.89). This demonstrates the feasibility and reliability of HT method in obtaining high-resolution permeability fields for predicting tracer spatial distribution.
(2) Laboratory experiments revealed and verified the enhanced transport effect of remediation agents under recirculating flow conditions in heterogeneous aquifers. The migration and distribution characteristics of the agents were clearly delineated. Results indicate that under enhanced hydraulic driving forces, tracers of identical initial concentrations can migrate farther. Even for distant monitoring points, observed concentrations under hydraulic driving can exceed those from higher dose stratified injections. Continuous water injection further amplifies agent transport under recirculating flow, significantly improving both spatial coverage and migration velocity.
(3) A numerical model of remediation agent spatiotemporal distribution under recirculating conditions was established based on HT method and validated using physical model results, confirming its reliability. The model elucidates the effects and mechanisms of factors such as extraction-injection well elevation difference, pumping rate, and aquifer heterogeneity on agent transport. Empirical relationships correlating these governing factors with extraction well concentrations were derived, providing actionable guidance for engineering applications.

This study not only furnishes a theoretical basis for integrating high-precision groundwater modeling into remediation strategy optimization but also bridges remediation design parameters with agent spatiotemporal distribution, supporting informed decision-making in field-scale engineering applications.

### Limitations of the study

It should be noted that this study still has several limitations: laboratory experiments should ideally employ authentic remediation agents rather than sodium chloride as tracers; the HT model demonstrates relatively low accuracy in characterizing heterogeneous aquifers at marginal zones of the sandbox; and challenges persist regarding constant-rate injection control. Both our research team and other investigators will continue to conduct more in-depth studies to address these limitations in future work.

## Resource availability

### Lead contact

Further information and requests for resources and reagents should be directed to and will be fulfilled by the lead contact, Huiyang Qiu (qiuhuiyang2023@163.com).

### Materials availability

This study did not generate new unique reagents.

### Data and code availability

• Data reported in this paper will be shared by the lead contact upon request.
• Code reported in this paper will be shared by the lead contact upon request.
• All other items reported in this paper will be shared by the lead contact upon request.

## Acknowledgments

This work was financially supported by the 10.13039/501100012166National Key Research and Development Program of China (grant no. 2023YFC3706005). H.Q. acknowledges the support from Nanjing Institute of Environmental Sciences specific fundings for basic scientific research (GYZX240409) and the fundamental research funds for the Central Universities (grant no. B250201288). R.H. acknowledges the support from the 10.13039/501100001809National Natural Science Foundation of China (NSFC grant no. 42372280).

## Author contributions

H.Q., methodology, data curation, and writing – original draft, review and editing; Z.Z.: conceptualization, validation, supervision, and writing – review and editing; C.N. and R.H., ideas, supervision, receive fundings, writing – review and editing; Y.S., methodology, data curation; L.W., J.H., and Y.Y., conceptualization, review, and editing.

## Declaration of interests

The authors declare no competing interests.

## STAR★Methods

### Key resources table

**Table:** 

REAGENT or RESOURCE	SOURCE	IDENTIFIER
**Software and algorithms**		

MMOC (finite-element code for variably saturated flow and solute transport)	Yeh et al. (Groundwater)^30^	https://doi.org/10.1111/j.1745-6584.1993.tb00597.x

### Experimental model and study participant

This study did not involve any experimental models or subjects typical in the life sciences. Therefore, this section is omitted.

### Method details

#### Constant head penetration test

The constant head permeability test is a common test method for determining the permeability coefficient of sand. The customized plexiglass permeameter (height 20 cm, diameter 7.5 cm) is used. The dried samples are layered into the soil column, and the water is slowly injected from bottom to top until saturation, maintaining the top liquid level overflow and maintaining a stable liquid level difference. The permeability coefficient is calculated according to Darcy‘s formula:

$$K=\frac{QL}{A\Delta h}=\frac{VL}{tA\Delta h}$$

In the formula, *K* is the permeability coefficient (m/s), *Q* is the flow rate (m^3^/s), *L* is the height of the permeameter (m), *A* is the cross-sectional area (m^2^), *Δh* is the head difference (m), *t* is the time (s).

#### Dispersion coefficient test

The one-dimensional soil column experiment is a critical method for studying the migration patterns of pollutants in groundwater. Its primary objective is to investigate the influence of porous media on pollutant transport in groundwater. This approach allows for the quantitative determination of solute transport parameters, the establishment of transport models aligned with real-world conditions, and the prediction of pollutant migration patterns. These results provide foundational experimental support for site simulations.

#### Experimental setup and parameters

The experiment was conducted in an organic glass soil column with a length of 20 cm and an inner diameter of 7.5 cm. A peristaltic pump controlled the injection rate of the tracer (NaCl solution). Three types of sand with distinct particle sizes were used to fill the column for one-dimensional dispersion experiments. Effluent samples were collected periodically at the outlet, and the conductivity of the solution was measured using a water quality analyzer. This process generated breakthrough curves (BTCs) for solute transport under varying flow rates and particle sizes.

#### Parameter calculation principles

For the one-dimensional soil column experiment, the governing equation is the one-dimensional advection-dispersion equation (ADE). Assuming a semi-infinite homogeneous sand column under steady-state flow conditions with an average pore velocity u, the mathematical model is expressed as:

$$\frac{\partial C}{\partial t}=D_{L}\frac{\partial^{2}C}{\partial x^{2}}-u\frac{\partial C}{\partial x}$$

Where C is the solute concentration (M/L^3^),t is time, *D*_*L*_ is the longitudinal dispersion coefficient (L^2^/T), and u is the average pore velocity (L/T).

The ADE model incorporates a transformation of the advection-diffusion equation by replacing the velocity u with the average velocity $\bar{u}$. Under steady-state injection conditions, the initial and boundary conditions are:

$$\begin{array}{ll} C\left(x,0\right)=0 & \left(x⩾0\right)\\ C\left(0,t\right)=C_{0} & \left(t>0\right)\\ C\left(\infty ,t\right)=0 & \left(t>0\right) \end{array}$$

The analytical solution to the ADE model is:

$$c\left(x,t\right)=\frac{c_{0}}{2}\left[\mathrm{erf}\;c\left(\frac{x-\bar{u}t}{2\sqrt{D_{L}t}}\right)+\text{ex}p\left(\frac{\bar{u}x}{D_{L}}\right)\mathrm{erf}\;c\left(\frac{x+\bar{u}t}{2\sqrt{D_{L}t}}\right)\right]$$

Where *c*_0_ is the initial solute concentration (mol/L).

Neglecting molecular diffusion, the longitudinal dispersion coefficient D_L_ is related to the seepage velocity u and dispersivity α by D_L_ = α*u* The longitudinal dispersion coefficient D_L_ was obtained by nonlinear least-squares fitting of the BTCs using Python, and the dispersivity was calculated for each sand type.

#### Parameters setup and analysis for simulation in discussion section

##### Depth of the extraction port

Existing studies have investigated the influence mechanisms of remedial reagent concentration and circulation operation mode on enhanced reagent transport, yet the selection and impact of well spatial positioning have rarely been discussed. To investigate the influence of different extraction port depths at the same location on enhanced migration effects, a series of tracer transport simulations (Case 1) were conducted based on the established numerical model with proven effectiveness. Specifically, the simulations demonstrated relative concentrations (*C*/*C*_0_) at four time ports (0, 26, 40, and 68 min) for tracer injection at a fixed location (port 11) with varying pumping port depths (port 8, 14, 20, and 26 from shallow to deep) (Figure 8). The red dot in the upper right represents the tracer injection port, while the black dots on the left indicate pumping ports. Taking the completion of tracer injection as the initial time (*t* = 0 min), concentration fields across different groups showed identical distributions, with tracers diffusing outward from the injection port and forming high-concentration zones only in proximity to the injection port, while exhibiting a leftward migration trend due to pumping effects. By 26 min, the tracer had expanded to a considerable area, with distinct concentration variations emerging among different pumping port groups. At 40 min, the diffusion range further expanded, and high-concentration zones began appearing near pumping ports. By 68 min, the distribution patterns and concentration profiles of tracers at some pumping ports reached a steady state, clearly indicating that high-concentration zones had arrived and were being extracted at the pumping ports.

The simulation results clearly demonstrate that different excavation port depths at the same location significantly affect tracer diffusion and enhanced migration effects. With a fixed shallow injection port (port 11), high-concentration zones had already bypassed the pumping ports in shallower ports (port 8, 14), whereas in deeper ports (port 20, 26), the diffusion range was smaller, and concentration distributions were more concentrated. This indicates that when excavation port depths are closer to the injection port, tracers diffuse faster and over a larger range, and vice versa. Additionally, the shape of tracer diffusion was influenced by excavation port depth: deeper ports (port 20, 26) resulted in a more uniform diffusion pattern with blurred boundaries, while shallower ports (port 8, 14) led to a more concentrated distribution with distinct, narrow boundaries. Thus, excavation port depth not only affects diffusion speed and range but also the morphology of tracer spread.

Beyond being controlled by excavation port depth, these simulation results were also influenced, albeit not dominantly, by aquifer heterogeneity, as evident from comparisons between port 8, 14, and 20 (Figures 8A–8C). Although both port 8 and port 20 belong to the same stratum (layer 1) as the injection port (port 11), significant differences in tracer diffusion speed, range, and morphology were observed between them. Conversely, port 14, though located in a different stratum compared to port 8 and 11, exhibited generally similar tracer migration trends. However, the higher peak concentration at 26 min, slower arrival of high-concentration zones to the excavation port by 40 min, and extended “tailing” effect at 68 min in port 14 relative to port 8 suggest lower permeability’s influence on tracer migration.

In conclusion: at the same port location, the closer the excavation port depth is to the injection port depth, the faster the tracer diffuses, the larger the spread, and the more uniform the distribution shape. Additionally, heterogeneous aquifer properties exert some influence on tracer migration rates. These findings hold substantial significance for optimizing tracer transport behavior and improving detection efficiency. By rationally selecting excavation port depths, migration pathways can be better controlled, ensuring both remediation efficiency and effectiveness at the site.

##### Depth of the injection port

Similarly, a series of tracer transport simulations were conducted using the port-established and highly effective numerical model to investigate the influence of different injection port depths at the same location on enhanced migration (Case 2). Specifically, the relative tracer concentrations (*C*/*C*_0_) were presented at four time ports (0, 26, 40, and 80 min) for varying injection port depths (ports 11, 17, 23, and 29, ordered from shallow to deep) with a fixed excavation port at port 26 (Figure 9). The red dot in the upper right represents the tracer injection port, while the black dot on the left indicates the pumping port, with the overall migration process being fundamentally consistent with the previous section. At the initial time (*t* = 0 min), corresponding to the end of tracer injection, concentration fields across different groups were identical, showing tracer diffusion outward from the injection port and forming high-concentration zones only near the injection location, with a leftward migration trend due to pumping effects. By 26 min, the tracer’s diffusion range expanded further, accompanied by notable changes in concentration distribution, indicating migration toward the pumping port. At 40 min, the diffusion range continued to increase, and concentrations near the pumping port began to rise, confirming progressive tracer movement toward extraction. By 80 min, both the distribution patterns and concentration profiles stabilized, clearly demonstrating significant tracer migration toward the pumping port.

The simulation results distinctly reveal that different injection port depths at the same location significantly influence tracer diffusion and enhanced migration. When a deeper injection port (port 11) was fixed, high-concentration zones in deeper injection ports (ports 23, 29) had already bypassed the excavation port, with port 29 exhibiting nearly complete tracer extraction. Conversely, shallower injection ports (ports 20, 26) showed smaller diffusion ranges and more concentrated distributions at the same time ports. This confirms that the closer the injection port depth is to the fixed pumping port depth, the faster the tracer diffuses and the broader its spread, and vice versa. Additionally, injection depth affected tracer diffusion morphology: deeper ports (ports 23, 29) produced sharper, narrower boundaries, while shallower ports (ports 11, 17) yielded more uniform distributions. Thus, injection port position governs not only diffusion speed and range but also the shape of tracer spread.

Analogous to the variable-pumping-port tests, the variable injection port simulations were also moderately influenced by aquifer heterogeneity, as evidenced by comparisons among ports 17, 23, and 29 (Figures 9B–9D). Though both ports 17 and 29 (injection ports) and the excavation port (port 26) belong to stratum 1, their tracer diffusion speeds, ranges, and morphologies diverged significantly. Meanwhile, injection port 23, despite being in a different stratum than ports 29 and 26, exhibited nearly identical migration trends. However, port 23’s higher peak concentration (26 min), delayed arrival of high-concentration zones at the pumping port (40 min), and prolonged “tailing” effect (80 min) highlighted the impact of lower permeability (port 23) on tracer transport. In summary, tracer diffusion accelerates and broadens with more uniform morphology as the injection port depth approaches the pumping port depth.

Notably, the initial concentration distributions differed markedly between variable-pumping-port (Case 1) and variable-injection-port (Case 2) tests. In Case 1, initial distributions were consistent across all pumping ports, with tracers diffusing radially from the injection port (the high-concentration zone) and exhibiting leftward migration tendencies. In contrast, Case 2 displayed pronounced initial disparities among injection ports, directly linked to formation heterogeneity. For ports 11 and 17 (located above layer 3, Figures 9A and 9B), pumping effects induced left-upward tracer flow around low-permeability zones. Meanwhile, ports 23 (within layer 2) and 29 (at layer 2’s edge, where pumping effects diminished) exhibited faster rightward flow due to higher hydraulic gradients on that side under Darcy’s law, resulting in broader right-side diffusion.

Crucially, combining results from both test types clarifies that tracer migration rates depend not on the absolute depths of pumping or injection ports but on their relative depths. Specifically, smaller relative depths (i.e., closer vertical separation) between pumping and injection ports correlate with faster diffusion, larger spread, and more uniform morphology. Strategic selection of injection and pumping port positions thus enables precise control over tracer migration pathways, enhancing both the accuracy and efficiency of soil and groundwater remediation efforts.

##### Pumping rate

Existing field-scale studies have demonstrated enhanced contaminant removal from groundwater through recirculation flushing (Song et al., 2021), yet the absence of quantified relationships between pumping/injection rates and remediation efficacy has constrained the implementation of precision remediation at contaminated sites. A series of tracer transport simulations were conducted using the port-established and high-performance numerical model to investigate the influence of different pumping rates on enhanced migration effects (Case 3). Specifically, the relative concentrations (*C*/*C*_0_) of the tracer, injected at a fixed location (port 11) and extracted at a fixed pumping port (port 26), were analyzed under varying pumping rates (8 mL/s, 12 mL/s, 16 mL/s, 24 mL/s, and 32 mL/s) at different time ports (85, 115, 170, 232, and 345 min) (Figure 10). The five sets of simulation images correspond to different pumping rates, visually demonstrating the relative concentration distribution of the tracer under varying temporal and spatial conditions.

The migration patterns across different pumping rate groups exhibited distinct characteristics: as the pumping rate increased from 8 mL/s to 32 mL/s, the tracer’s diffusion range expanded overall, but the expansion exhibited nonlinear behavior. At lower pumping rates (8–16 mL/s), the tracer plume predominantly migrated along the direct path between the injection and pumping ports, preferentially forming dominant flow channels through high-permeability zones. However, when the pumping rate increased to 24–32 mL/s, enhanced hydraulic gradients caused the tracer to spread into intermediate- and low-permeability zones surrounding the pumping port, resulting in a broadening plume morphology with noticeable lateral dispersion, particularly in intermediate-permeability regions. Permeability heterogeneity significantly influenced local migration speeds. The tracer front advanced most rapidly in high-permeability zones, forming high-concentration core areas (corresponding to relative concentration values of 0.6–0.8). Intermediate-permeability zones exhibited slower diffusion, while low-permeability zones displayed pronounced retention effects, maintaining concentrations mostly in the range of 0.2–0.4. Under low pumping rates (Figures 10A–10C), almost no migration was observed in low-permeability zones, with only minimal penetration detectable at high pumping rates (Figures 10D–10E).

The experimental results demonstrate that tracer migration in heterogeneous aquifers is significantly governed by the coupling effects of pumping rate and formation permeability. Higher pumping rates increase hydraulic gradients, intensifying advective transport and enabling the tracer to overcome permeability barriers. In contrast, permeability variations dictate local flow velocities and preferential migration paths by altering effective porosity and hydraulic conductivity. Low-permeability zones, due to high capillary resistance and limited effective flow paths, significantly slow tracer front propagation, resulting in lower accumulated concentrations. Intermediate-permeability zones form transitional diffusion zones under elevated hydraulic gradients, while high-permeability zones consistently dominate the primary migration pathways. It is noteworthy that as pumping rates increase, the capture efficiency of high-permeability zones diminishes (plume widening reduces core concentrations), while contributions from intermediate- and low-permeability zones gradually rise.

These findings collectively indicate that tracer migration in heterogeneous aquifers exhibits strong path dependence, shaped simultaneously by pumping intensity and permeability heterogeneity. At low pumping rates, migration is dominated by high-permeability pathways, displaying pronounced directional selectivity. In contrast, higher pumping rates enhance driving forces, weakening the influence of permeability barriers and promoting tracer dispersion into intermediate- and low-permeability zones, thereby achieving broader aquifer volume coverage, a crucial consideration for optimizing contaminant extraction in groundwater remediation strategies.

##### Quantitative analysis

The previous analyses demonstrate that the regional tracer concentration field undergoes dynamic adjustments in response to variations in pumping well depth, injection well depth, pumping rate, and aquifer heterogeneity. However, the temporal evolution of concentrations at key monitoring points, particularly extraction wells, holds greater practical significance for remediation applications. Therefore, systematic analyses were conducted to characterize the evolution of relative tracer concentrations (*C*/*C*_0_) at pumping wells in heterogeneous aquifers with respect to migration time (*t*), elevation difference (*δ*H) between pumping and injection wells, and pumping rate (*v*) (Figure S5).

The 3D scatterplot (Fig. 11a) reveals distinct concentration response patterns across monitoring points: steep rising limbs and rapid peak attainment (*C*/*C*_0_ ≈ 0.8) were observed at locations like Port 8, whereas Port 26 exhibited gradual rises with delayed, diminished peaks (*C*/*C*_0_ < 0.04). These morphological variations primarily stem from the coupled effects of elevation difference (*δ*H) and spatial positioning—monitoring points in high-permeability zones with minimal *δ*H (e.g., Port 8) demonstrated advection-dominated transport with sharp, high-concentration breakthrough curves, whereas those in intermediate- and low-permeability zones or with large *δ*H (e.g., Port 26) displayed diffusion-controlled migration with broad, low-amplitude, lagging curves. Notably, all breakthrough curves followed a consistent pattern: rapid ascent to peak concentration followed by slow decay (Figure S5B), though decay rates varied spatially due to permeability-dependent tracer retention. The heterogeneous curve shapes reflect nonlinear, site-specific concentration dynamics. Multi-linear regression yielded empirical time-concentration expressions with satisfactory goodness-of-fit (*R*^2^ > 0.6, mean >0.7), enabling predictive applications; the outlier poor fit for Port 20 likely relates to stratum 3’s low permeability.

Peak concentration analysis across scenarios revealed an inverse correlation between *δ*H and both concentration magnitude and time-to-peak (solid line, Figure S5A). Projections onto three orthogonal planes elucidated multidimensional relationships, with strong linear trends emerging between *δ*H versus time-to-peak (*R*^2^ = 0.87) and *δ*H versus peak concentration (*R*^2^ = 0.67). These high-fidelity empirical relationships provide robust tools for spatiotemporal concentration prediction: The fitted equations effectively capture dominant transport mechanisms while accounting for permeability-induced heterogeneity in arrival timing and concentration attenuation.

(Equation 7) CC0max=−0.65δH+0.24

(Equation 8) t(CC0max)=11400δH+2100

Similarly, the analysis of simulation results (fixed pumping well, variable injection wells) reveals marked differences between experimental groups. Clusters corresponding to lower-elevation injection wells (e.g., Ports 23 & 29) concentrate in shorter migration times (<500 s) with peak *C*/*C*_0_ exceeding 0.75, while higher-elevation groups (e.g., Ports 11 & 17) exhibit prolonged migration (>1000 s) with diminished peak concentrations (<0.4) (Figure S5C). Distinct curve morphologies emerge: lower injection wells yield steep ascents (time-to-peak <200 s) with sharp maxima (*C*/*C*_0_ ≈ 0.8), whereas higher wells produce broad plateaus (time-to-peak >600 s) with blunt peaks (*C*/*C*_0_ ≈ 0.35) (Figure S5D). Fitted concentration-time empirical expressions demonstrate reasonable predictive capability (*R*^2^ > 0.55, mean >0.65) for pumping well concentrations.

Projection analysis of peak concentrations across scenarios onto three orthogonal planes confirms linear relationships between elevation difference (*δ*H) and both time-to-peak (positive correlation) and peak concentration magnitude (negative correlation). These trends reflect physical retardation and hydrodynamic dispersion effects along extended migration pathways. High-fidelity empirical relationships (0.71 & 0.68) quantitatively capture these spatiotemporal distribution patterns:

(Equation 9) CC0max=−1.46δH+0.52

(Equation 10) t(CC0max)=10350δH+885

This dichotomy originates from injection well positioning relative to permeability architecture. Proximal injection wells coupled with high-permeability pathways enable rapid tracer advection (short transit times, limited dilution), whereas distal wells force circuitous routing through low-permeability zones, extending migration paths while enhancing diffusion-driven attenuation (50% *C*/*C*_0_ reduction). These mechanisms provide critical insights for predicting contaminant trajectories and remediation timelines in heterogeneous aquifers. Parallel analysis simulations (Figure S5E) demonstrates pumping rate (*v* = 8–32 mL/s) modulation of *C*/*C*_0_ temporal evolution. High-rate clusters (*v* ≥ 24 mL/s) form steep cones (*C*/*C*_0_ > 0.75) at short timescales (*t* < 3000 s), while low-rate groups (*v* ≤ 12 mL/s) spread as subdued platforms (*C*/*C*_0_ = 0.3–0.5) beyond 6000 s. Robust concentration-time models (R^2^ > 0.75, mean >0.8) support operational predictions.

(Equation 11) CC0max=−0.0007v+0.064

(Equation 12) t(CC0max)=−294.18v+10603

XY-plane projections (*R*^2^ = 0.84) confirm that increased *v* reduces time-to-peak by enhancing hydraulic gradients and preferential advection. Conversely, XZ-plane trends (*R*^2^ = 0.94) reveal paradoxical peak suppression at higher rates, accelerated transport causes premature concentration decline, potentially compromising remediation efficacy. These quantifiable rate-dependent spatiotemporal patterns establish a verifiable framework for optimizing extraction-injection parameters in heterogeneous groundwater systems.

### Quantification and statistical analysis

Tracer concentrations were calculated from conductivity using a linear calibration. Spatial distributions of hydraulic head and relative concentration (C/C_0_) were generated by kriging interpolation. Model performance was evaluated by comparing predicted versus observed hydraulic heads and concentrations. The coefficient of determination (R^2^) was calculated for calibration (eight pumping tests plus extraction-injection data) and validation (eight pumping tests). For solute transport simulations, predicted and observed concentrations at monitoring ports were compared, and R^2^ values were reported for each test group. Empirical relationships linking peak concentration (C/C_0_max) and time-to-peak at extraction wells to elevation difference (δH) between wells and to pumping rate (v) were derived by multi-linear regression. Goodness-of-fit (R^2^) for each regression model is provided in the main text. All data are presented as raw measurements or interpolated fields, with R^2^ values used as measures of predictive accuracy.

### Additional resources

No additional resources were generated during this study.
