# Supplementary material for: Enhanced remediation of contaminated aquifers by circulating flow field: Laboratory sandbox with quantitative analysis
Source: iScience. 2026 Jul 9;29(8):116237. doi: 10.1016/j.isci.2026.116237 (PMC13380121; doi:10.1016/j.isci.2026.116237)
Supplement: Document S1. Figures S1–S6 and Tables S1–S3 [file mmc1.pdf]

iScience, Volume *29*

Supplemental information

Enhanced remediation of contaminated aquifers by circulating flow field: Laboratory sandbox with quantitative analysis

Huiyang Qiu, Ziwen Zhou, Lei Wang, Jing Hua, Chicgoua Noubactep, Yang Song, Yizhi Yuan, and Rui Hu

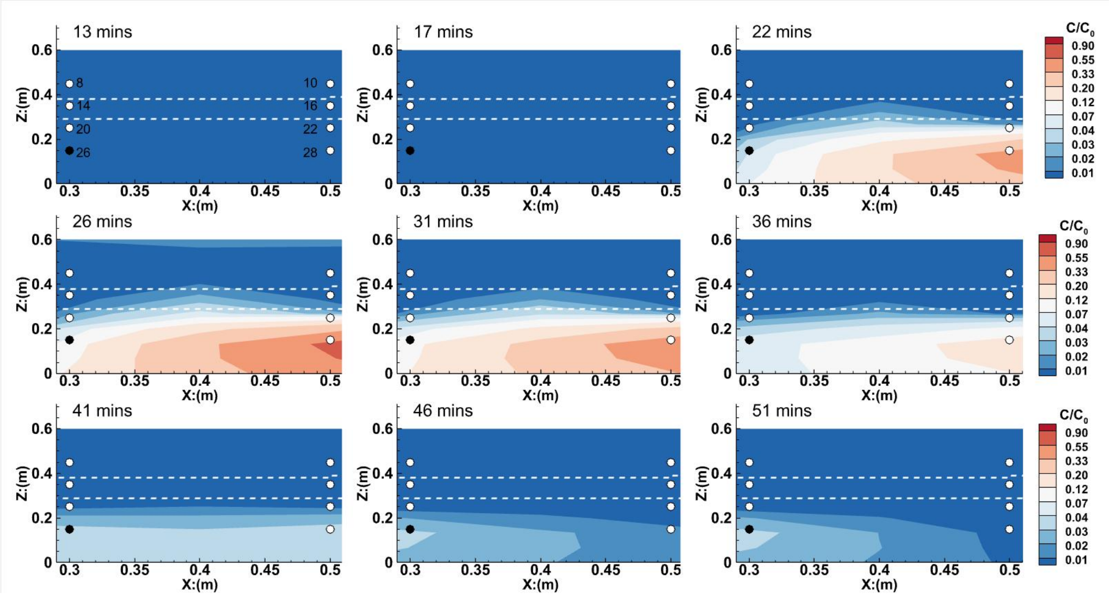

Fig. S1 The concentration distribution diagram with time of laboratory excavation-injection experiment (Test 1)

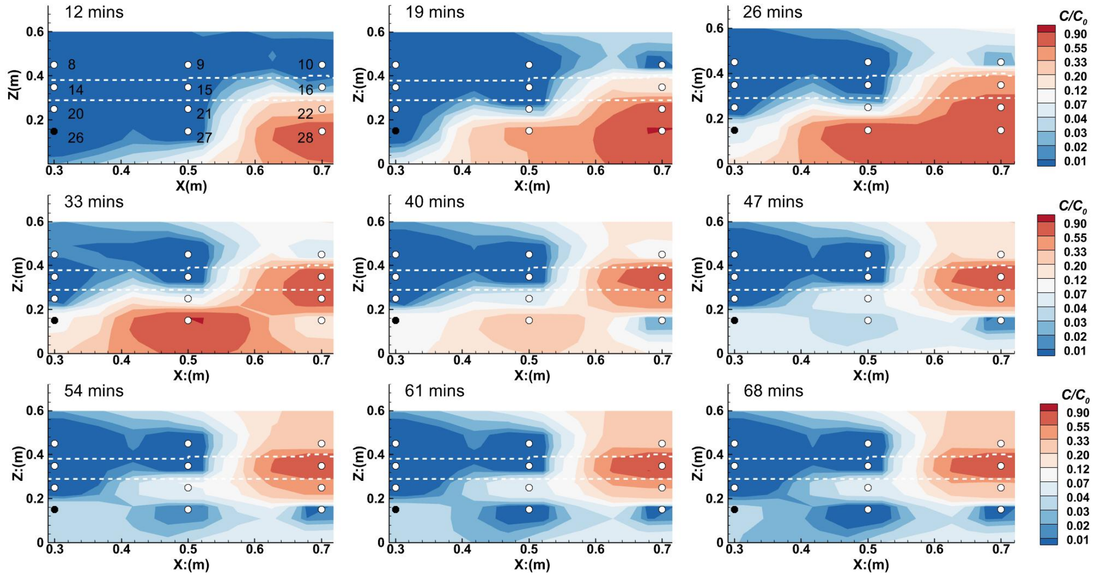

Fig. S2 The concentration distribution diagram with time of laboratory excavation-injection experiment (Test 4)

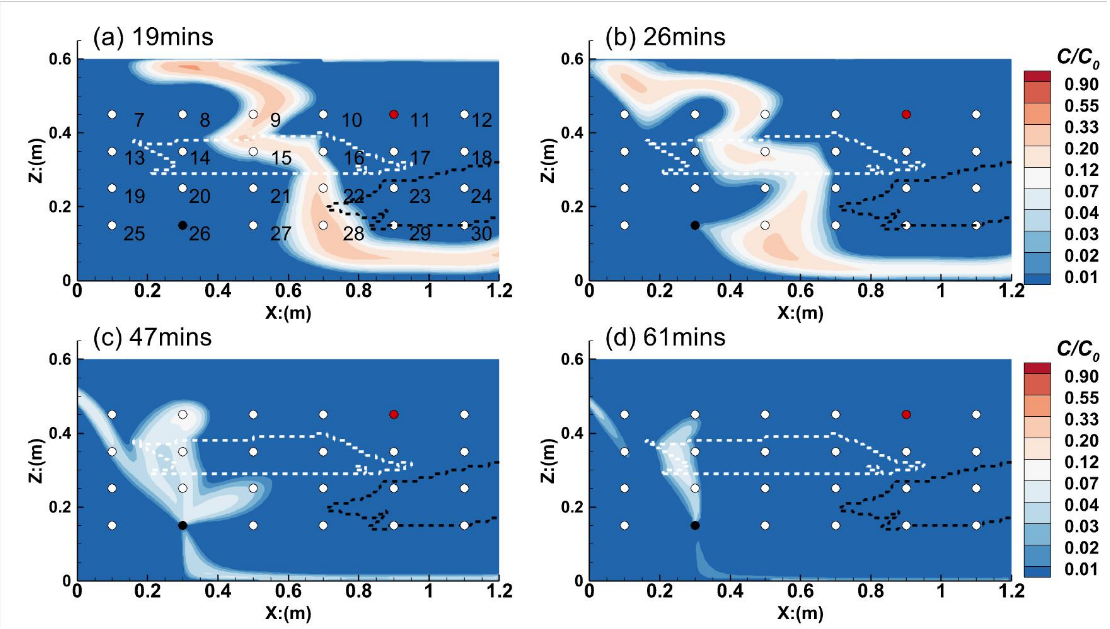

Fig. S3 The concentration distribution diagram with time of modeled excavation-injection experiment (Test 3)

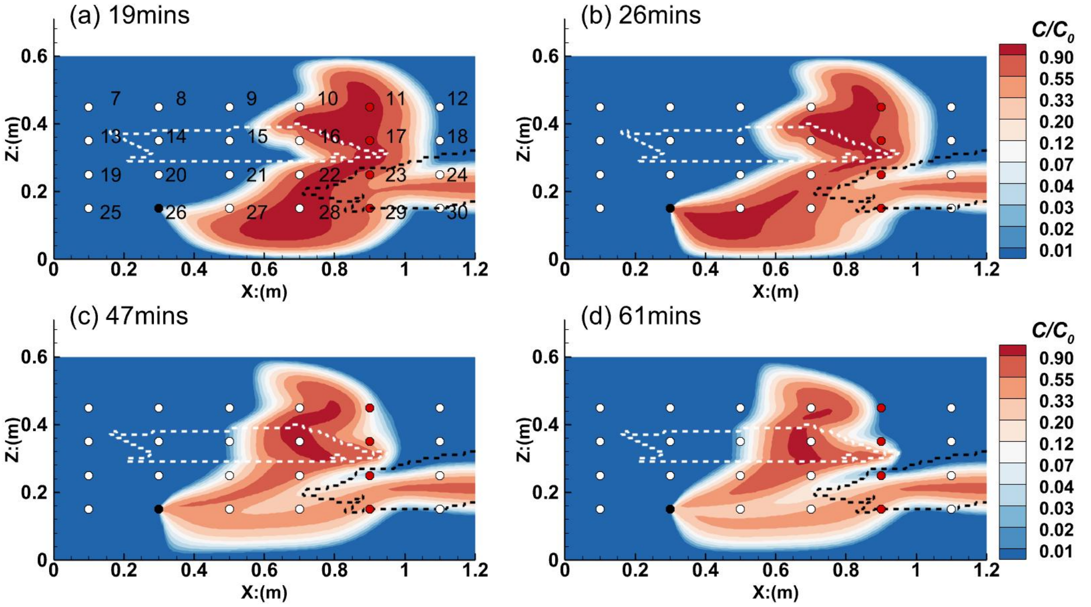

Fig. S4 The concentration distribution diagram with time of modeled excavation-injection experiment (Test 4)

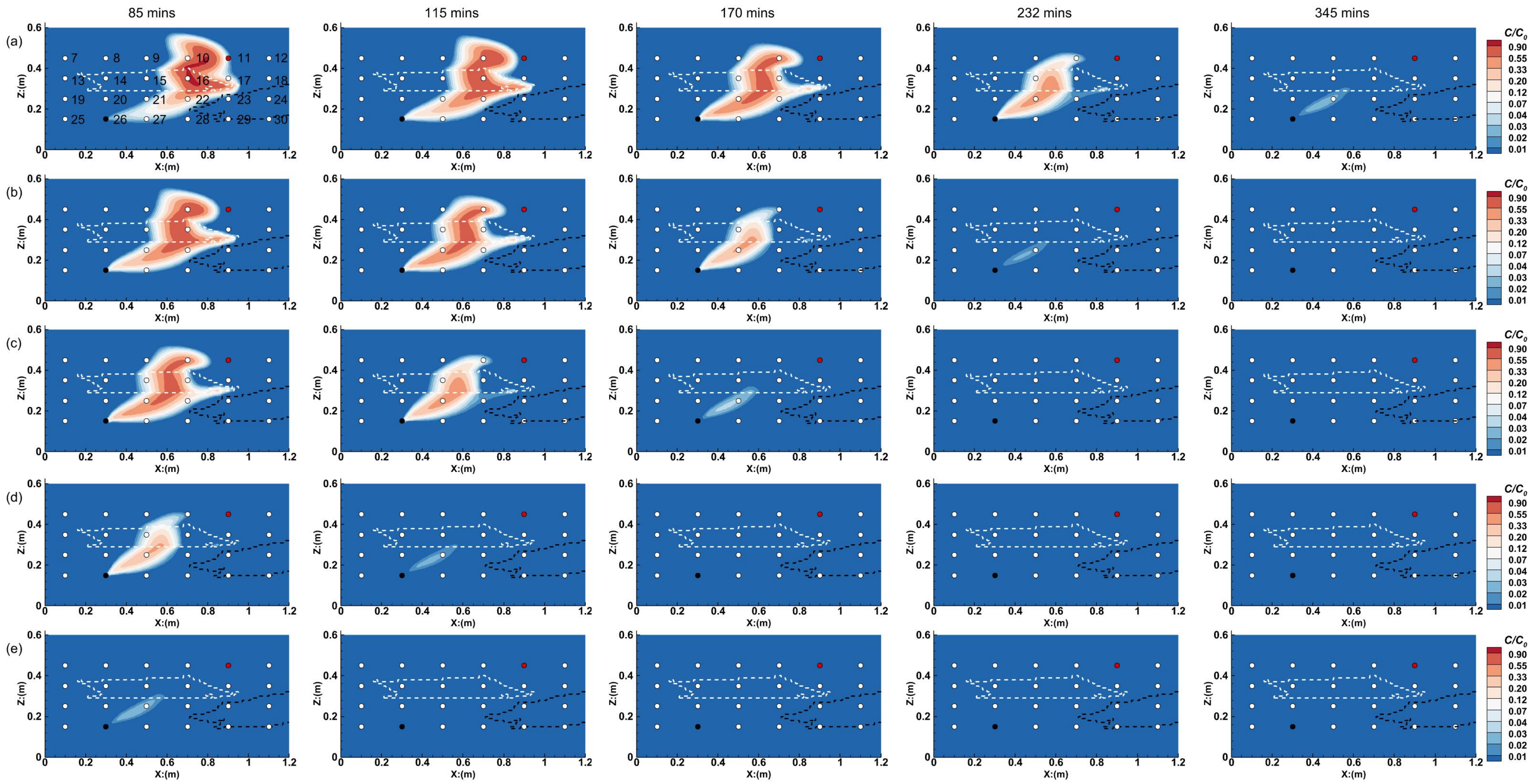

Fig. S5 Simulation result for Case 3 using various pumping rate :(a) 8 ml/s, (b) 12 ml/s, (c) 16 ml/s, (d) 24 ml/s, (e) 32 ml/s. Red dots represent injection ports, and black dots represent extraction port.

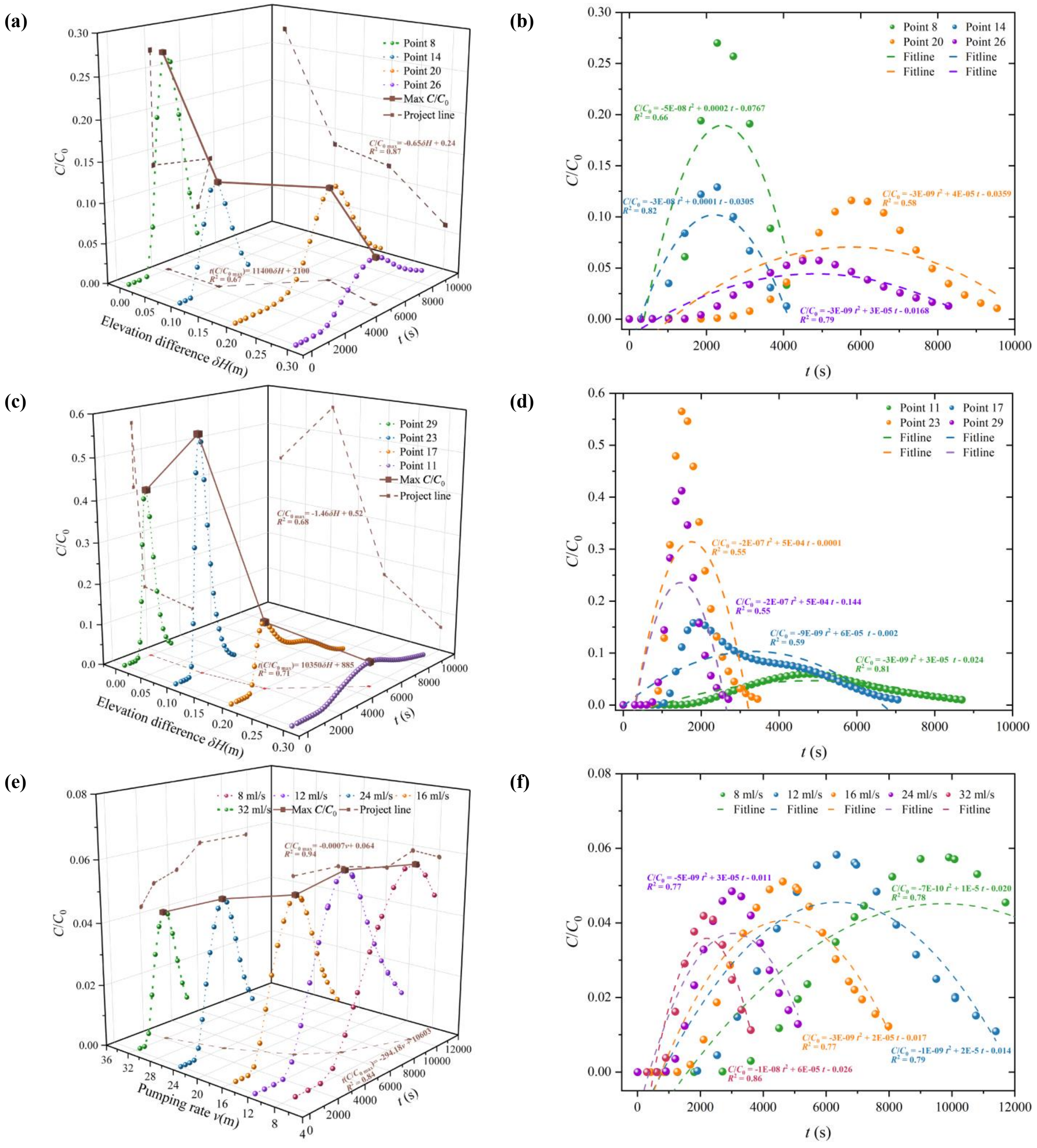

Fig. S6 Relative concentration variations at extraction ports under different operational conditions versus. (a) depth difference and time with variable pumping well; (b) time with variable pumping well; (c) depth difference and time with variable injection well; (d) time with variable injection well; (e) depth difference and time with variable pumping rates; (f) time with variable pumping rates.

Table S1 Parameter values for three filled sandy soil layers with various particle size

Layers	particle size (mm)	specific gravity	porosity	permeability coefficient (cm/s)
1	2~3	2.65	0.47	0.438
2	0.2~0.5	2.64	0.4	0.0733
3	<0.08	2.63	0.3	0.00351

Table S2 Experimental design details

Tests	Extraction port	Injection port	NaCl injection concentration (mg/L)	Injection volume (ml)	Continuously inject water	Pumping rate (ml/s)	Injection rate (ml/s)
Test1	26	29	7000	1800	No	16.064	23.077
Test2	26	11	9000	1800	No	16.064	23.077
Test3	26	11	9000	1800	Yes	16.064	25
Test4	26	11, 17, 23, 29	9000	7200	No	16.064	23.077

Table S3 Pumping test parameters

Pumping port	Aquifer	Pumping rate (ml/s)	Pumping port	Aquifer	Pumping rate
8	2	21.65	20	2	16.10
9	2	24.63	21	2	18.33
10	2	27.00	22	2	11.31
11	2	18.00	23	1	20.37
14	3	19.60	26	2	26.70
15	3	11.05	27	2	22.00
16	3	8.25	28	2	22.00
17	2	19.70	29	1	30.00
